# Trends in risk factors for coronary heart disease in the Netherlands

**DOI:** 10.1186/s12889-016-3526-7

**Published:** 2016-08-19

**Authors:** C. Koopman, I. Vaartjes, A. Blokstra, W. M. M. Verschuren, M. Visser, D. J. H. Deeg, M. L. Bots, I. van Dis

**Affiliations:** 1 Julius Center for Health Sciences and Primary Care, University Medical Center Utrecht (STR 6.131), P.O. Box 85500, 3508 GA Utrecht, The Netherlands; 2 Dutch Heart Foundation, The Hague, The Netherlands; 3 National Institute for Public Health and the Environment, Bilthoven, The Netherlands; 4 Department of Health Sciences, EMGO+ Institute, VU University, Amsterdam, The Netherlands; 5 Department of Dietetics and Nutrition Sciences, Internal Medicine, VU University Medical Center, Amsterdam, The Netherlands

**Keywords:** Coronary heart disease, Risk factors, the Netherlands, Trends

## Abstract

**Background:**

Favourable trends in risk factor levels in the general population may partly explain the decline in coronary heart disease (CHD) morbidity and mortality. Our aim was to present long-term national trends in established risk factors for CHD.

**Methods:**

Data were obtained from five data sources including several large scale population based surveys, cohort studies and general practitioner registers between 1988 and 2012. We applied linear regression models to age-standardized time trends to test for statistical significant trends. Analyses were stratified by sex and age (younger <65 and older ≥65 years adults).

**Results:**

The results demonstrated favourable trends in smoking (except in older women) and physical activity (except in older men). Unfavourable trends were found for body mass index (BMI) and diabetes mellitus prevalence. Although systolic blood pressure (SBP) and total cholesterol trends were favourable for older persons, SBP and total cholesterol remained stable in younger persons.

**Conclusions:**

Four out of six risk factors for CHD showed a favourable or stable trend. The rise in diabetes mellitus and BMI is worrying with respect to CHD morbidity and mortality.

**Electronic supplementary material:**

The online version of this article (doi:10.1186/s12889-016-3526-7) contains supplementary material, which is available to authorized users.

## Background

Non-communicable diseases (NCD) are the main cause of death worldwide. In the Netherlands, 90 % of deaths are related to the main four NCDs (cancers 33 %, cardiovascular diseases 29 %, chronic respiratory diseases 6 % and diabetes 2 %) [1]. Both modifiable risk factors (behavioural) and non-modifiable risk factors (such as age, gender, ethnicity, family history) can change the risk for certain NCDs. Coronary heart disease (CHD) is a major component of cardiovascular deaths. Systolic blood pressure (SBP), cholesterol levels, body mass index (BMI), smoking, diabetes and physical inactivity are major risk factors for NCDs, but especially for cardiovascular disease. CHD incidence, hospitalization, case-fatality and mortality rates have markedly decreased in the Netherlands in the last decades, as in other Western societies [2–4]. Declines in CHD mortality could be attributed to improved medical treatments and favourable changes in modifiable cardiovascular risk factors, which can be attained by an increase in the use of cardiovascular drugs and revascularization procedures and by improvements in lifestyle [5]. Previous international research suggests that 25–50 % of the CHD mortality decline can be explained by changes in medical treatments and the remaining 50–75 % by changes in modifiable risk factors [6–8].

Monitoring cardiovascular risk factor trends is of great importance as favourable changes in cardiovascular risk factors can cause declines in CHD incidence rates, short-term case-fatality rates (by less severe events), risks for re-infarctions and mortality rates. Previous health policies and strategies can be evaluated by monitoring parallel changes in risk factors. Furthermore, valuable information on past and current trends can help to design future interventions and address future policy questions.

Favourable trends for physical activity and smoking have been observed for the Dutch population [9, 10], whereas no consistent trends in cholesterol levels were seen [11]. Furthermore, substantial rises in the prevalence of obesity, diabetes and small increases in blood pressure levels have also been observed, which might attenuate the previously observed decline in CHD incidence, hospitalization and mortality rates [12–14]. However, the time span that was covered by previous observations was outdated and fragmented. Furthermore, older adults (over 70 years) were not included, despite that the largest burden of CHD is in this age group. We aimed to provide a descriptive analysis of age-sex specific time trends in systolic blood pressure (SBP), body mass index (BMI), total cholesterol levels, diabetes mellitus, physical activity and smoking prevalence in the general population including ages up to 85 years between 1988 and 2012.

## Methods

Data were obtained from five data sources including cohort studies, surveys and general practitioner registers, that were representative for the Dutch population. Characteristics of the five data sources that we used are presented in Table 1.

**Table 1:** Characteristics of the five data sources used in this study

Data source	Risk factor	Study design	Response rate	*N*	Age range	Time period
Doetinchem Cohort Study	SBP <65 years Total cholesterol <65 years BMI <65 years	Prospective cohort study	Baseline 62 % follow-up 75–80 %	7769 at baseline	20–59 Years at baseline	1987–2007
LASA	SBP ≥65 years Total cholesterol ≥65 years BMI ≥65 years	Prospective cohort study	Baseline 55–60 % follow-up 85 %	3107 at baseline	55–85 Years at baseline	1995–2009
STIVORO	Smoking	Survey	~70 % overall	Sample ~20,000 each year	Aged 15 years and over	1988–2011
HNU^a^	Diabetes mellitus	GP Register/Dynamic cohort study	n/a	70,854 in 1996 100,359 in 2012	All ages included	1996–2012
CBS Gezondheidsenquete	Physical activity	Survey	60–65 % Overall	Sample ~15,000 each year	Aged 18 years and over	2001–2011

*SBP* systolic blood pressure, *BMI* body mass index, *LASA* Longitudinal Aging Study Amsterdam, *STIVORO* Stichting Volksgezondheid en Roken, *HNU* Huisartsen register Utrecht, *GP* general practitioner, *CBS* Centraal Bureau voor de Statistiek

^a^GP register Continue Morbiditeits Registratie-Nijmegen, GP register Registratienet Huisartsenpraktijken-Limburg, the Doetinchem Cohort Study and LASA were used to adjust diabetes prevalences from HNU (see Additional file 1)

### Data sources

#### Systolic blood pressure (SBP)

Data on SBP were obtained from the National Institute for Public Health and the Environment (RIVM) Doetinchem Cohort study for individuals under 65 years of age [15]. The Doetinchem Cohort study started in 1987. During the period 1987–1991 baseline data was obtained from 7,769 individuals, aged 20–59 years at baseline. The study comprised a physical examination for measurements of body weight, height, systolic and diastolic blood pressure, a non-fasting blood sample (total cholesterol) and questionnaire information on lifestyle and diet. The overall response rate at baseline was 62 %. Follow-up examinations were carried out every 5 years, with response rates varying between 75 and 80 %. Blood pressure was measured twice in each examination in sitting position after 2 min of rest. The mean value of two measurements was used in the analyses. Blood pressure in examination 4 was measured with a different device and patients sat in a slightly different position. Random coefficient analyses were used to obtain a correction factor for examination 4 to make blood pressure values at the different examinations comparable [16].

For individuals aged 65 years or above we used data from the Longitudinal Aging Study Amsterdam (LASA) [17]. LASA is an ongoing study focusing on physical, emotional, cognitive, and social functioning in older adults. Data collection started in 1992, from the population registries of 11 municipalities in the Netherlands. During the period 1992–1993 baseline data was obtained from 3,107 individuals, aged 55–85 years. Every examination consisted of two parts, a main examination and a medical interview. The initial response rate was 60 %. Follow-up examinations were carried out every 3 years since then. The response rates for follow-up measurements were around 85 %. In 2002–2003, a new cohort was sampled (*n* = 1,002, 55–65 years). The initial response rate of the new cohort was 55 %. Both samples were combined for the present analyses. Blood pressure was measured in sitting position, three times in the first examination, once in examination 2 and 3, three times in examination 4 and 5 and four times in examination 6. The mean value of the measurements was used in the present analyses. Blood pressure in the first examination was measured with a different device than in the other examinations. Therefore, we only included data on blood pressure of examinations 2 to 6.

#### Total cholesterol

Data on total cholesterol was obtained from the Doetinchem Cohort study for individuals aged 35–64 years and from LASA for individuals aged 65 years or over. Total cholesterol was measured in all 4 examinations in the Doetinchem Cohort Study, until 1998 in non-fasting EDTA-plasma and from 1998 onwards in serum at a lipid reference laboratory, using standardized enzymatic methods. Total cholesterol was measured in examination 2 and 6 in LASA, in non-fasting EDTA plasma samples stored at −80 °C, using an enzymatic (CHOD-PAP) colorimetric test.

#### BMI

Data on BMI was obtained from the Doetinchem Cohort Study for individuals aged 35–64 years and from LASA for individuals aged 65 years and over. Weight and height of participants in the Doetinchem Cohort Study were measured with participants wearing light indoor clothing with emptied pockets and without shoes. Body weight was measured to the nearest 0.1 kg on calibrated scales and height to the nearest 0.5 cm. BMI in the Doetinchem-study was calculated as weight minus 1 kg to adjust for clothing, divided by height squared (kg/m^2^). Weight and height of participants in LASA were measured with participants wearing underwear. Some participants wore clothes or a corset during the measurement. The adjustment for clothing in LASA was minus 1 kg for clothing and/or minus 1 kg for a corset.

#### Smoking

Smoking data was obtained from the Dutch expert centre on tobacco control STIVORO (Stichting Volksgezondheid en Roken, STIVORO, TNS-NIPO Continue Onderzoek Rookgewoonten (COR) 1988–2011). COR collects online-data throughout the year of 20,000 adults aged 15 years and over about the number of people who smoke, the characteristics of smokers and smoking behaviour. The response rate was 70 % in 2011. The question in the survey we used was “Do you smoke (sometimes)?” (yes/no). This particular question has not changed during the study period.

#### Diabetes mellitus

Diabetes mellitus data was obtained from the general practitioner (GP) registry ‘Huisartsen Netwerk Utrecht’ (HNU). The registry data were collected from 1996 to up to 2012. HNU is a GP registry of 5 GP practices comprising about 100,000 patients in 2012. We defined diabetes mellitus as an International Classification in Primary Care (ICPC)-code T90 mentioned in the electronic patient record. Information was collected from Huisartsen Informatie Systeem data (HIS: GP information system). Since the information on diabetes mellitus dealt with previous or current diagnosis of diabetes mellitus, the time window for patients in the beginning of the registry was shorter than for those who were entered later in the registry. As a consequence, the estimates of diabetes mellitus prevalence in the beginning years of the study period were underestimated. To adjust for underestimation, we created an adjustment factor based on the average relative increase in diabetes prevalence between 1997 and 2007 that we estimated using all available data sources (GP register Continue Morbiditeits Registratie-Nijmegen, GP register Registratienet Huisartsenpraktijken-Limburg, the Doetinchem Cohort Study and LASA). More information on the calculation of the adjustment factor can be found in the Additional file 1.

#### Physical activity

The ‘Centraal Bureau voor de Statistiek Gezondheidsenquete’ is a health survey using a questionnaire on various health aspects performed in inhabitants of the Netherlands. Information was available on physical activity from 2001 to 2011 from about 15,000 individuals each year. The response rates varied between 60 and 65 %. Physical activity was defined as self-reported compliance to the Dutch physical activity standard entitled ‘Norm gezond bewegen’. Individuals of 18 to 54 years of age were classified as active when they participated in activities with a Metabolic Equivalent of Task (MET) ≥4 for at least 30 min a day for at least 5 days a week. Individuals aged 55 years or over were classified as active when they participated in activities with a Metabolic Equivalent of Task (MET) ≥3 for at least 30 min a day for at least 5 days a week.

### Data analyses

We applied linear regression models to the age-standardized trends to investigate whether trends over time were statistically significant. Statistical significance was based on a two-sided P-value less than 0.05. It was not possible to calculate P-values for trends in total cholesterol ≥65 years because we only had two measurement points available. Trends in risk factors were presented separately for men and women and standardized to the age distribution of the Dutch population of 2010. Standardisation was done using a direct method with 10 year age groups. Trends in continuous risk factors (SBP, total cholesterol and BMI) were presented as age-standardized mean values. To calculate 95 % confidence intervals (CI) for continuous risk factors we used 1.96 times the standard error (SE) of the mean either side of the estimate. Trends in dichotomous risk factors (smoking, diabetes and physical activity) were presented as prevalence proportions (%). To calculate 95 % CI for dichotomous risk factors we used 1.96 times the SE either side of the estimate, where

$$ \mathsf{S}\mathsf{E}=\sqrt{\frac{\mathsf{p}\left(\mathsf{1}\hbox{-} \mathsf{p}\right)}{\mathsf{n}}} $$

Factor p comprises the prevalence proportion of the risk factor and factor n the sample size. Data were analysed using SPSS version 20 and Microsoft Excel 2007.

## Results

**Fig. 1:**
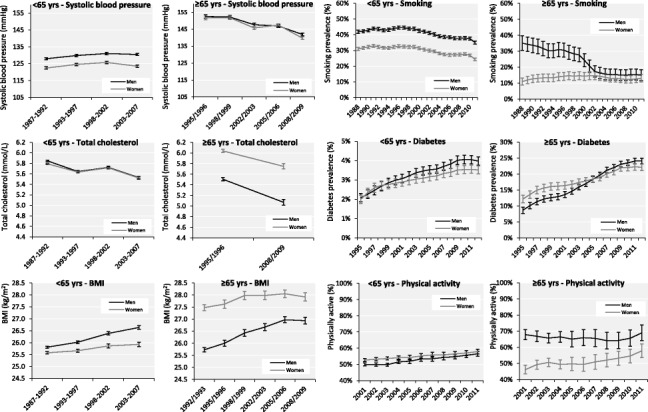
Age-standardized sex-specific time trends in risk factors for coronary heart disease in adults <65 years and elderly ≥65 years in the Netherlands

Age-sex specific trends are presented in Fig. 1. Results are summarized in Table 2.

**Table 2:** Dutch time trends in cardiovascular risk factors: main findings of the analyses

	Adults <65 years		Elderly ≥ 65 years	
	Men	Women	Men	Women
SBP	=	=	↓	↓
Total cholesterol	=	=	↓	↓
BMI	↑	↑	↑	=
Smoking	↓	↓	↓	=
Diabetes	↑	↑	↑	↑
Physical activity	↑	↑	=	↑

*SBP* systolic blood pressure, *BMI* body mass index

↑, increasing time trend. ↓, decreasing time trend. =, stable time trend

### SBP

Between 1987–1992 and 2003–2007, SBP remained stable over time in persons <65 years, with fluctuation in men between 127.9 mmHg and 131.1 mmHg (*P* = 0.15), and in women between 122.4 mmHg and 125.7 mmHg (*P* = 0.59). In persons ≥65 years, SBP declined in men from 152.5 mmHg in 1995/1996 to 141.9 mmHg in 2008/2009 (*P* < 0.01) and in women from 151.7 mmHg to 139.7 mmHg (*P* = 0.04).

### Total cholesterol

Between 1987–1992 and 2003–2007, total cholesterol remained stable over time in men and women <65 years with fluctuation between 5.5 mmol/l and 5.8 mmol/l (*P* = 0.15 in men and *P* = 0.19 in women). In persons ≥65 years, total cholesterol declined in men from 5.5 mmol/l in 1995/1996 to 5.1 mmol/l in 2008/2009 and in women from 6.0 mmol/l to 5.7 mmol/l.

### BMI

Between 1987–1992 and 2003–2007, BMI increased in men <65 years from 25.8 kg/m^2^ to 26.6 kg/m^2^ (*P* < 0.01) and in women from 25.6 kg/m^2^ to 25.9 kg/m^2^ (*P* = 0.02). In persons ≥65 years, BMI increased in men from 25.7 kg/m^2^ in 1992/1993 to 26.9 kg/m^2^ in 2008/2009 (*P* = 0.001) and remained stable in women from 27.5 kg/m^2^ to 27.9 kg/m^2^ (*P* = 0.052).

### Smoking prevalence

Between 1988 and 2011, the prevalence of smoking decreased in men <65 years from 42 to 35 % (*P* < 0.001) and in women from 31 to 24 % (*P* < 0.001). In persons ≥65 years, the prevalence of smoking decreased in men from 35 % in 1988 to 15 % in 2011 (*P* < 0.001) and remained stable over time in women around 11–13 % (*P* = 0.75).

### Diabetes mellitus prevalence

Between 1995 and 2012, the prevalence of diabetes increased in men <65 years from 2.1 to 4.0 % (*P* < 0.001) and in women from 2.0 to 3.5 % (*P* < 0.001). In persons ≥65 years, the prevalence of diabetes increased in men from 8.7 % in 1995 to 24.1 % in 2012 (*P* < 0.001) and in women from 12.2 to 22.1 % (*P* < 0.001).

### Physical activity

Between 2001 and 2011, the proportion that complied to the Dutch physical activity standard increased in men <65 years from 50 to 56 % (*P* < 0.001) and in women from 53 to 58 % (*P* < 0.001). In persons ≥65 years, the proportion that complied to the Dutch physical activity standard remained stable over time in men around 68–69 % (*P* = 0.64) and increased in women from 46 to 58 % (*P* < 0.001).

## Discussion

This study adds important information about long-term national trends in CHD risk factors in the Netherlands between 1988 and 2012. The results demonstrated favourable trends in smoking (except in women ≥65 years) and physical activity (except in men ≥65 years). Unfavourable trends were found for BMI and diabetes prevalence. Although SBP and cholesterol trends were favourable for those aged 65 years or over, SBP and cholesterol remained stable in men and women <65 years.

Our results are consistent with results from several other studies performed in Western societies during approximately the same time frame. Declines in smoking and cholesterol levels have been apparent in most western European countries [18–23]. Increases in body mass index and diabetes mellitus have been reported in many established and emerging economies [6, 8, 23–25]. Information on adherence to the physical activity recommendation is more limited, but generally shows improvement over time [21, 22]. In countries surrounding the Netherlands decreasing trends in SBP been reported across all age groups [18, 19, 21, 23, 26]. Despite the similarity of the other risk factor trends in these countries, we did not observe a decline in SBP in persons aged 65 years or below.

There are several factors and health policies that could have contributed to the observed trends in cardiovascular risk factors: i) nationwide campaigns to raise awareness and promote healthy behaviour (primary prevention), ii) screening and case-finding in high risk populations (primary prevention), iii) cardiovascular risk management in patients and high-risk groups following the most recent guidelines (primary and secondary prevention), iv) government policies, measures, restrictions and changes in for example product availability or content (primary prevention).

Decreasing trends in blood pressure and cholesterol, especially in older people, may be due to the increase in use of blood pressure and cholesterol lowering drugs [5]. A nationwide salt campaign started in 2006 (at the end of our study period) by the Dutch Consumer Organisation and salt content in bread was reduced by legislation. However, analyses of the Doetinchem Cohort Study showed that the salt intake in persons under 65 years did not decline between 2006 and 2010 [27]. The UK initiated a nationwide salt reduction programme in 2003/2004. As expected, the observed declines in SBP at population-level are larger in the UK than in the Netherlands [21, 23]. The increase in BMI may explain part of the lack of decline in systolic blood pressure in those under 65 years, and is also likely to explain the increase in diabetes. Although total fat intake decreased, saturated fat intake remained quite stable [28]. Data from Dutch food consumption surveys (VCPs) showed that the intake of trans fatty acids has decreased over time, from 4.5 % in 1987/1988 to 0.5 % in 2007/2010 in the Netherlands, a trend that may have affected the population total cholesterol level beneficially [29, 30]. The dramatic increase in diabetes prevalence likely reflects a combination of an increase in new cases and more active screening for diabetes by general practitioners resulting in more undetected diabetes mellitus patients being identified.

The Dutch government has formulated several objectives in the last decades, aimed at diminishing the problem of obesity, but none of these objectives have been reached. Nevertheless, this study showed that the increase in BMI in older women levelled off. Several nationwide campaigns to raise awareness on overweight and promote physical activity were organised during the study period. An evaluation of a large 5-year mass media campaign (2002–2007) on overweight and physical activity showed that despite the high levels of free publicity and campaign exposure, the campaign was not strong enough to provoke substantial changes in important predictors of behaviour change [31]. Joint efforts of the government, food industry, retail and NGO’s since 2005 resulting in a covenant on overweight are promising.

The decline in smoking may in part be attributed to the increasing awareness and education level in the Netherlands on the adverse effects of smoking. Furthermore, the Dutch expert centre on tobacco control STIVORO took the lead in launching an intensive media-supported millennium smoking cessation campaign. It was estimated that 600.000 people (>15 % of smokers) attempted to quit smoking at the millennium and 12 % managed to abstain for more than 1 year. Smoke-free legislation started in the Netherlands in 2004 with a workplace smoking ban and in 2008 with the hospitality industry ban. An evaluation study observed a decline in smoking prevalence after the implementation of the workplace ban, but not after the hospitality industry smoking ban [32]. Furthermore, tobacco tax increases (2001, 2004, and 2008) were implemented in the Netherlands and likely have contributed to the decline in smoking. A systematic review showed that a 10 % increase in price increased cessation rates in adults with 3–5 % [33].

Our study is the first to show an overview of Dutch time trends in all major risk factors for CHD, including young and older adults. Ideally, all risk factors would be measured repeatedly over time in one large cohort or survey that is representative for the general population. Unfortunately, data on risk factors is scattered in the Netherlands, and thus we had to use the best data available from different data sources. The size, quality and methodology of these studies differed. Trends in SBP, cholesterol and BMI were estimated by repeated measurements within one cohort. Response rates varied over time, which may have resulted in biased trend estimates. Yet previous research with similar data showed limited selective dropouts [34]. Nevertheless, absolute levels of risk factors could be different from the general population since those who volunteered to take part in studies tend to be healthier than those who do not. Absolute differences in risk factors over time could be smaller in healthier cohorts compared to the general population. Moreover, lower baseline levels of risk factors will leave less room for improvement. Also, self-reporting could have underestimated absolute levels of smoking and overestimated physical activity levels. This misclassification is expected to be constant over time and therefore not influencing our trend analyses.

Trends in risk factors are generally used for evaluating public health policies and interventions and to estimate the changes in CHD risk in a population. In the Netherlands, we recently showed a steep decline in incidence of acute myocardial infarction, a major contributor to CHD. From 1998 to 2007 the decline in incidence was 38 % in men and 32 % in women [2]. Although BMI and diabetes showed unfavourable trends, the steep decline in incidence of acute myocardial infarction suggests that the balance between favourable and unfavourable trends goes in favour of CHD risk reduction. However, the poorer health of the younger generation is a rising threat for the current decline in cardiovascular disease and therefore monitoring of population-wide risk factor levels is highly warranted [34]. We advise to integrate and harmonize the different Dutch data sources from cohorts, surveys and registers.

## Conclusion

In conclusion, four out of six risk factors for CHD showed a favourable or stable trend. The rise in diabetes mellitus and BMI is worrying. These trends need to be monitored and addressed by interventions to ensure a continuing decline in CHD morbidity and mortality.

## Additional files

Additional file 1: Diabetes mellitus measurements and corrections. (PDF 207 kb) Additional file 2: Ethics and consent to participate and Data availability and materials. (PDF 220 kb) Additional file 3: Smoking raw data. (XLSX 12 kb) Additional file 4: Physical activity raw data. (XLSX 15 kb)

## Abbreviatons

BMI: Body mass index
CBS: Centraal Bureau voor de Statistiek
CHD: Coronary heart disease
CI: Confidence interval
COR: Continue Onderzoek Rookgewoonten
EDTA: Ethylenediaminetetraacetic acid
GP: General practitioner
HIS: GP information system
HNU: Huisartsen Netwerk Utrecht
ICPC: International Classification in Primary Care
LASA: Longitudinal Aging Study Amsterdam
MET: Metabolic equivalent of task
NCD: Non-communicable diseases
RIVM: National Institute for Public Health and the Environment
SBP: Systolic blood pressure
SE: Standard error
SPSS: Statistical package for the social sciences
STIVORO: Stichting Volksgezondheid en Roken
TNS-NIPO: Taylor Nelson Sofres Nederlands Instituut voor Publieke Opinie
UK: United Kingdom
VCPs: Dutch food consumption surveys
