# Supplementary material for: Trends in risk factors for coronary heart disease in the Netherlands
Source: BMC Public Health. 2016 Aug 19;16:835. doi: 10.1186/s12889-016-3526-7 (PMC4992244; doi:10.1186/s12889-016-3526-7)
Supplement: Additional file 1: — Diabetes mellitus measurements and corrections. (PDF 207 kb) [file 12889_2016_3526_MOESM1_ESM.pdf]

Supplementary file 1. Trends in risk factors for coronary heart disease in the Netherlands

Diabetes mellitus measurements and corrections

Diabetes mellitus data was obtained from the general practitioner (GP) registry 'Huisartsen Netwerk Utrecht' (HNU). HNU is a GP registry of 5 GP practices comprising around 60,000 patients in 1996. The registry data were collected from 1996 to up to 2012. We defined diabetes mellitus as an International Classification in Primary Care (ICPC)-code T90 mentioned in the electronic patient record. Information was collected from Huisartsen Informatie Systeem data (HIS: GP information system). Since the information on diabetes mellitus dealt with previous or current diagnosis of diabetes mellitus, the time window for patients in the beginning of the registry was shorter than for those who were entered later in the registry. As a consequence, the estimates of diabetes mellitus prevalence in the beginning years of the study period were underestimated. To adjust for underestimation, we created an adjustment factor based on the average relative increase in diabetes prevalence between 1997 and 2007 that we estimated using all available data sources (GP register Continue Morbiditeits Registratie-Nijmegen, GP register Registratienet Huisartsenpraktijken-Limburg, the Doetinchem Cohort Study and LASA).

We did not have age-specific data available from GP registers Continue Morbiditeits Registratie-Nijmegen and Registratienet Huisartsenpraktijken-Limburg. The number of diabetics in the Doetinchem Cohort Study was relatively small, therefore we chose to use HNU as main data source (table 1, table 2). We increased the prevalence of diabetes in HNU in 1997 by applying this adjustment factor which was 2.23 in men <65 years, 2.30 in women <65 years, 1.98 in men ≥65 years and 2.69 in women ≥65 years (table 3). We gradually increased the adjustment factors by every year before 2007 in a log-linear way, so that the relative increase in diabetes prevalence between 1997 and 2007 equaled the calculated relative increase from the combined data sources (table 4).

Table 1. Trends in diabetes mellitus prevalence in available data sources

Data source diabetes mellitus	Sample size study (n)	Trend 1997 - 2007	
		Men	Women
HNU GP register	100.000 in 2007	+ 252%	+ 248%
RNH-Limburg GP register	87.000 in 2005	+ 60%	+40%
CMR-Nijmegen GP register	13.500 in 2007	+ 75%	+33%
Doetinchem cohort study (<65 years)	3.600 in 2007	- 2%	- 14%
LASA cohort (≥65 years)	1.500 in 2007	+ 114%	+19%

HNU, Huisartsen Netwerk Utrecht. RNH, Registratienet Huisartspraktijken Limburg. CMR, Continue Morbiditeits Registratie,. LASA, Longitudinal Aging Study Amsterdam.

Table 2. Mean trend in diabetes mellitus prevalence between 1997-2007 by age-sex group

Age-sex group	Data sources used	Mean trend 1997 – 2007
Men <65 years	HNU, RHN, CMR, Doetinchem	+ 44%
Men ≥65 years	HNU, RHN, CMR, LASA	+ 83%
Women <65 years	HNU, RHN, CMR, Doetinchem	+ 20%
Women ≥65 years	HNU, RHN, CMR, LASA	+ 31%

Table 3. Age-sex specific adjustment factors diabetes mellitus prevalence for HNU values 1997

Age-sex group	Trend in HNU 1997 – 2007	Mean trend 1997 – 2007	Adjustment factor data 1997 (trend in HNU / mean trend)
Men <65 years	+ 222%	+ 44%	3.22 / 1.44 = 2.23
Men ≥65 years	+ 262%	+ 83%	3.62 / 1.83 = 1.98
Women <65 years	+ 175%	+ 20%	2.75 / 1.20 = 2.30
Women ≥65 years	+ 253%	+ 31%	3.53 / 1.31 = 2.69

Table 4. Example correction factors diabetes mellitus prevalence men <65 years.
We gradually increased the adjustment factors by every year before 2007 in a log-linear way, so that the relative increase in diabetes prevalence between 1997 and 2007 equaled the calculated relative increase from the combined data sources.

Year	Correction factor for HNU values
1993	3.08
1994	2.84
1995	2.62
1996	2.42
1997	2.23
1998	2.06
1999	1.90
2000	1.75
2001	1.62
2002	1.49
2003	1.38
2004	1.27
2005	1.17
2006	1.08
2007	1
2008	1
2009	1
2010	1
2011	1
2012	1
