# Supplementary material for: Trends in risk factors for coronary heart disease in the Netherlands
Source: BMC Public Health. 2016 Aug 19;16:835. doi: 10.1186/s12889-016-3526-7 (PMC4992244; doi:10.1186/s12889-016-3526-7)
Supplement: Additional file 2: — Ethics and consent to participate and Data availability and materials. (PDF 220 kb) [file 12889_2016_3526_MOESM2_ESM.pdf]

Supplementary file 2. Trends in risk factors for coronary heart disease in the Netherlands

Table 1 Summary of ethics and consent to participate for the data sources whose data we used.

Data source	Study design	Ethics and consent to participate	Data availability and materials
Doetinchem Cohort Study	Prospective cohort study	Informed consent was obtained by the principal investigators. The Medical Ethics Committee (METC) of the Netherlands Organization for Applied Scientific Research Institute (93/01) and the METC of the University Medical Center Utrecht (07/233) approved the Doetinchem Cohort Study.	The data used in this study is non-public data and will not be shared. This data is only available for authorized researchers. For access to the data or review purposes approval should be obtained by the principal investigator of the data source (for more information http://www.rivm.nl).
LASA	Prospective cohort study	Informed consent was obtained by the principal investigators. The Medical Ethical Exam of the VU University Medical Centre approved the LASA Study (92/139, 2002/141).	The data used in this study is non-public data and will not be shared. This data is only available for authorized researchers. For access to the data or review purposes approval should be obtained by the principal investigator of the data source (for more information http://www.lasa-vu.nl)
STIVORO	Survey	Ethics approval and consent was not applicable for this survey.	Data was available from http://www.stivoro.nl. We included the raw data in Supplementary file 3 Smoking.
HNU[1]	GP Register / Dynamic cohort study	Ethics approval and consent was not applicable for this GP register.	The data used in this study is non-public data and will not be shared. This data is only available for authorized researchers. For access to the data or review purposes approval should be obtained by the principal investigator of the data source (for more information http://portal.juliuscentrum.nl/nl-nl/home.aspx)
CBS Gezondheidsenquete	Survey	Ethics approval and consent was not applicable for this survey.	Data was available from http://statline.cbs.nl. We included the raw data in Supplementary File 4 Physical activity.

LASA, Longitudinal Aging Study Amsterdam. STIVORO, Stichting Volksgezondheid en Roken. HNU, Huisartsen register Utrecht. GP, general practitioner. CBS, Centraal Bureau voor de Statistiek.

[1] GP register Continue Morbiditeits Registratie-Nijmegen, GP register Registratienet Huisartsenpraktijken-Limburg, the Doetinchem Cohort Study and LASA were used to adjust diabetes prevalences from HNU (see Appendix)
